# Assembled genomic and tissue-specific transcriptomic data resources for two genetically distinct lines of Cowpea ( *Vigna unguiculata* (L.) Walp)

**DOI:** 10.12688/gatesopenres.12777.2

**Published:** 2018-06-18

**Authors:** Andrew Spriggs, Steven T. Henderson, Melanie L. Hand, Susan D. Johnson, Jennifer M. Taylor, Anna Koltunow

**Affiliations:** 1 CSIRO Agriculture and Food, Acton, ACT, 2601, Australia; 2 CSIRO Agriculture and Food, Urrbrae, SA, 5064, Australia; 3 University of Adelaide, Adelaide, SA, 5000, Australia

**Keywords:** cowpea, genome, transcriptome, male and female gametogenesis, seed

## Abstract

Cowpea ( *Vigna unguiculata* (L.) Walp) is an important legume crop for food security in areas of low-input and smallholder farming throughout Africa and Asia. Genetic improvements are required to increase yield and resilience to biotic and abiotic stress and to enhance cowpea crop performance. An integrated cowpea genomic and gene expression data resource has the potential to greatly accelerate breeding and the delivery of novel genetic traits for cowpea. Extensive genomic resources for cowpea have been absent from the public domain; however, a recent early release reference genome for IT97K-499-35 ( *Vigna unguiculata* v1.0, NSF, UCR, USAID, DOE-JGI, http://phytozome.jgi.doe.gov/) has now been established in a collaboration between the Joint Genome Institute (JGI) and University California (UC) Riverside. Here we release supporting genomic and transcriptomic data for two transformable cowpea varieties, IT97K-499-35 and IT86D-1010. The transcriptome resource includes six tissue-specific datasets for each variety, with particular emphasis on reproductive tissues that extend and support the *V. unguiculata* v1.0 reference. Annotations have been included in our resource to allow direct mapping to the v1.0 cowpea reference. The resource described here is supported by downloadable raw and assembled sequence data.

## Introduction

Cowpea ( *Vigna unguiculata* (L.) Walp) is a versatile grain legume crop, also cultivated for vegetative consumption and animal fodder. The grain provides a rich source of protein (25% by weight) for human consumption. Cowpea was domesticated in sub-Saharan Africa and is relatively resilient to heat and drought stress. It has the ability to fix atmospheric nitrogen, and cowpea is often intercropped with cereals or used in crop rotations. Cowpea is grown frequently on subsistence and smallholder farms in mixed crop-livestock systems, particularly in low-input farming systems in the semi-arid regions of West and Central Africa, South America, and Asia ( Singh, 2014). Cowpea is a vital component for nutrient security in global agricultural communities.

Cowpea crop improvement has been led by the International Institute of Tropical Agriculture (IITA) through the generation of multiple varieties with improved yield and stress tolerance. However, further improvement is required as many varieties in use exhibit low yield, disease susceptibility, and are prone to abiotic stress ( Hall, 2012). Reproductive characteristics have been revisited in cowpea recently and developmental calendars developed for two cowpea varieties developed by IITA, IT86D-1010 and IT97K-499-35 together with supporting developmental experimental tools to support seed yield improvements ( Salinas-Gamboa *et al.,* 2016). One approach to increase yield aims to alter sexual reproductive development in high yielding hybrids to an asexual mode in order to assess if it is feasible to save hybrid cowpea seed each growing season ( Salinas-Gamboa *et al.,* 2016; Capturing Heterosis OPP1076280). Technological advances in genetic profiling and DNA sequencing approaches over the last decade have facilitated the recent establishment of genomic resources for cowpea ( Muñoz-Amatriaín *et al*., 2017). These data resources have the potential to rapidly accelerate cowpea crop improvement through molecular assisted breeding, characterisation of population diversity and various genomic editing technologies.

The cowpea genome (2 *n*=22) has an estimated size of 620 megabases (Mb) ( Chen *et al*., 2007). Analyses of cDNA libraries from 17 different cowpea accessions were used to identify 183,118 expressed sequence tags (ESTs) and 29,728 ‘unigene’ sequences ( Muchero *et al*., 2009). Subsequently, high-throughput sequencing and EST-derived single nucleotide polymorphisms (SNPs) have formed the basis for rapid improvement in consensus genetic maps for cowpea ( Lucas *et al*., 2011; Muchero *et al*., 2009; Muñoz-Amatriaín *et al*., 2017). The current consensus map contains 37,372 SNP loci mapped to 3,280 bins and spans 837.11 cM with sub-centimorgan average density (0.26 cM) ( Muñoz-Amatriaín *et al*., 2017).

Most genomic characterisation to date has focussed on the cowpea variety IT97K-499-35, adapted for West Africa. A substantial new genomic resource for IT97K-499-35 containing 97,777 assembled DNA contigs of greater than 1 kb in length, representing 323 Mb of the cowpea genome, has been recently released ( Muñoz-Amatriaín *et al*., 2017). This assembly was combined with sequencing data from two genomic bacterial artificial chromosome (BAC) libraries to generate a BAC physical map ( Muñoz-Amatriaín *et al*., 2017). Despite the substantial contribution and utility of these resources, they did not represent a complete contiguous sequence or ‘reference’ assembly of the cowpea genome.

University California Riverside (UCR) in collaboration with the Joint Genome Institute have since generated an early release of an annotated genome reference for cowpea (IT97K-499-35) ( ***Vigna unguiculata*** NSF, UCR, USAID, DOE-JGI. https://phytozome.jgi.doe.gov/pz/portal.html#!info?alias=Org_Vunguiculata_er). This resource incorporates long-read sequence technology enabling the assembly of 519.4 Mb into 11 pseudo-molecules and 722 scaffolds generated by UC Riverside and subsequently annotated by the Joint Genome Institute. When finalised, this resource will be foundational to future advances in cowpea crop improvement and will serve as an important unified resource for cowpea crop research.

In this publication, we describe and release survey genome assemblies and tissue-specific transcriptome assemblies derived from IT86D-1010 and IT97K-499-35 to supplement and extend the existing cowpea sequence resources. These cowpea varieties, of different pedigrees, are transformable using *Agrobacterium*-mediated gene insertion ( Popelka *et al.,* 2006). They therefore represent important genetic resources for investigating and substantiating gene function. In addition, their genomic and transcriptomic characterisation will enable identification and testing of cell-type specific promoters and genic tools that should facilitate the examination and synthesis of reproductive pathways to improve seed yield in cowpea. We have therefore developed transcriptomic resources to characterise expressed genes in leaf and importantly floral tissues undergoing male and female gametogenic development, and early seed initiation.

The survey genome assembly of IT97K-499-35 supports the reference genome assembly, *Vigna unguiculata* v1.0, of IT97K-499-35; however, the IT86D-1010 data resource is the first public genome-scale resource for this variety. Additional cowpea transcriptome resources are provided for leaf and reproductive tissues for both IT86D-1010 and IT97K-499-35. In accordance with the policies of early release genomes, an extensive comparative analysis of data provided here with the reference assembly ( *Vigna unguiculata* v1.0) is not provided. However, we have annotated our transcriptomic and genomic contig data with coordinates of the v1.0 reference, based on IT97K-499-35, to further facilitate integration of publicly available cowpea genome and transcriptome resources.

Transcriptomes of multiple tissues derived from IT97K-499-35 have been generated and previously published ( Yao *et al.,* 2016; http://vugea.noble.org). Tissues previously profiled were predominately vegetative and included leaf, stem, root and flower from 5-week-old plants, empty seed pods at 6, 10 and 16 days after pollination and seeds at 8, 10, 14 and 18 days after pollination (DAP) ( Yao *et al.,* 2016). In this publication, we provide the first transcriptomic characterisation in both IT97K-499-35 and IT86D-1010 for floral tissues undergoing male and female gametogenic development, and early seed initiation.

The work described in this publication provides a unique and valuable extension to emerging genomic and transcriptomic resources in cowpea. These foundational resources will enable identification and testing of cell-type specific promoters and genic tools that should facilitate the examination and synthesis of reproductive pathways to improve seed yield in cowpea. All transcriptomic and genomic resources are provided with coordinate-based annotation to the IT97K-499-35 reference genome ( *V. unguiculata* v1.0) providing integration of these resources to assist coordinated scientific progression of the cowpea research community.

## Methods

### Plant materials and tissue collection

Cowpea lines IT86D-1010 and IT97K-499-35 were originally sourced from the International Institute of Tropical Agriculture (IITA) and their pedigrees are provided in Supplementary Figure 1. Lines have been maintained in CSIRO for more than 10 generations (not through single seed descent). Material (IT97K-499-35) used in the generation of the reference assembly ( *Vigna unguiculata* v1.0) was sourced from Mike Timko at the School of Medicine, University of Virginia, who had previously received the material from IITA. UC Riverside took the IT97K-499-35 line through single seed descent and confirmed 100% homozygosity before bulking. Analysis is underway to compare the CSIRO lines and UC Riverside lines to quantitatively assess genetic similarity of the independently sourced seed stocks. The plants were grown as described by Salinas-Gamboa *et al*. (2016). Young unexpanded leaves were collected for DNA and total RNA extraction for both lines. The reproductive calendars developed for these varieties by Salinas-Gamboa *et al*. (2016) were used to harvest a set of five reproductive tissue types from both IT86D-1010 and IT97K-499-35. Anther tissues containing developing male gametophytes at pollen mother cell, tetrad and mature bicellular pollen stages were pooled to form a pooled male gametophyte (PMG) sample for both lines. In addition, ovules were extracted from both lines from floral buds to provide individual tissue samples containing differentiated megaspore mother cells (MMCs), female meiotic tetrads (FMT), and mature female gametophytes (MFG) at anthesis. Finally, early developing seeds (ES) were collected post-fertilization containing a mixture of zygotes and early globular embryos with proliferating endosperm.

### Nucleic acid extraction and sequencing

DNA and RNA extractions were carried out using a Qiagen maxi DNA kit and Qiagen RNeasy plant mini kit, respectively, as per the manufacturer’s instructions. Illumina library preparation and sequencing of DNA and RNA was undertaken by the Australian Genome Research Facility (AGRF) with 2 × 100 bp standard insert paired-end sequencing using a Hiseq 2500 system. Shotgun sequencing libraries from single IT86D-1010 and IT97K-499-35 genomic DNA samples were prepared using the Illumina TruSeq Nano DNA Library Prep Kit as per the manufacturer’s instructions. Whereas the Illumina TruSeq Stranded mRNA Library Prep Kit was used to prepare poly(A) mRNA sequencing libraries from total RNA samples as per the manufacturer’s instructions. Three replicate libraries were prepared and sequenced for each of the RNA samples except for the IT97K-499-35 MMC and FMT samples, where two replicates were sequenced.

### Sequence analysis

Raw genomic DNA sequencing reads from IT86D-1010 and IT97K-499-35 were separately assembled into contigs using Biokanga (version 4.3.6) in a multi-step process. First, raw reads were run through ‘biokanga filter’, where common adapter, primer, and vector contaminants were identified and trimmed. Redundant copies of identical paired-end read pairs were removed, and pairs with no sequence overlap to other raw sequence were also removed as they provided no value to the assembly. Filtered paired-end reads were then assembled into contigs, using ‘biokanga assemb’, with default parameterisation that allows 1 base substitution per 100bp of sequence overlap. Resulting contigs were run through a second ‘reassembly’ step with ‘biokanga assemb’, allowing up to 5 base substitutions per 100bp of sequence overlaps to provide reduction in redundant sequence representations. Finally, ‘biokanga scaffold’ added ordering to some contigs, by identifying raw paired-end pairs that match to ends of different contigs under assumptions of sequencing insert fragment size of 110–1500bp. Raw tissue-specific RNASeq reads were separately assembled into transcriptome contigs using Biokanga, with the same multi-step process as used for the genomic DNA reads (above), without the reassembly step to retain putative transcript isoforms. Summary quality metrics for all DNA and RNA sequence reads are provided in the data repository associated with this paper as Supplementary Data 1. These metrics include read length, average GC content and average quality score across the length of the read, the read midpoint and read end.

The assembled genomic DNA sequences of IT86D-1010 and IT97K-499-35 were annotated for predicted gene regions using Augustus v3.1.0 ( Stanke & Waack, 2003). From the available Augustus training sets, tomato ( *Solanum lycopersicum,* ITAG2.4) gene sequences were selected in Augustus on the basis of the greater percentage of cowpea RNA reads covered by the resulting gene predictions. Predictions from the Augustus approach also encompassed gene predictions from both DNA strands, partial gene predictions and predictions of untranslated regions (UTRs). The resulting protein sequence predictions, with a minimum length of 100 amino acids, were annotated through matches to the NCBI’s ‘nr’ protein sequence database (downloaded 8 ^th^ August 2017) using ‘blastp’ with an e-value threshold of 1e-50. 

To complete sequence alignment analysis, the genomic DNA sequencing reads and the tissue-specific RNASeq reads from IT86D-1010 and IT97K-499-35 were pre-processed by ‘biokanga filter’ as described above, prior to alignment with the genomic sequence assemblies of IT86D-1010 and IT97K-499-35 and to the *Vigna unguiculata* v1.0 reference genome sequences. The software ‘biokanga align’ was used for these alignments and unique-best alignments for each paired-end sequence with an insert fragment size of 100–1000bp to a genomic sequence were reported, with at most 3 base substitutions per 100bp. Auto-end-trimming (read chimera detection) was permitted to 50bp where required. Detection and reporting of SNPs between DNA or RNA sequencing reads and assembled genomes was enabled where there was coverage of at least 5 reads.

## Dataset validation

### Genomic sequence data for IT86D-1010 and IT97K-499-35

A total of 527 and 303 million pair-end DNA sequence reads from IT86D-1010 and IT97K-499-35, were generated, respectively. These were assembled into 39,123 contigs for IT86D-1010 and 57,690 contigs for IT97K-499-35 with average lengths of 15.6 and 9.8 kilobases (kb), respectively ( Table 1). The contig assemblies generated were able to incorporate 68–73% of the raw DNA reads generated ( Table 2). The majority (>87%) of the assembled genomic contigs from IT86D-1010 and IT97K-499-35 could be mapped to the *V. unguiculata* v1.0 reference genome ( Table 3) with a minimum of 70% contig overlap. When the required contig overlap increased to 90%, contig mapping to the reference decreased to an average of 63% across assembled datasets. Possible causes for lack of mapping at higher stringencies are loss of contiguous alignment or loss of fidelity of assembly towards the end of the survey assembly contigs. *In-silico* gene prediction identified approximately 60,000 putative coding sequences in both IT86D-1010 and IT97K-499-35 and nearly 70% of these could be annotated to published protein sequences within the NCBI nr public database ( Table 4).

**Table 1.** Details of IT97K-499-35 and IT86D-1010 genomic DNA contigs generated and assembled in this study. Contigs of less than 1000 base pairs were excluded in this summary. Comparison to the *V. unguiculata* genome v1.0 of IT97K-499-35 is provided.

	IT97K-499-35 gDNA ^1^	IT86D-1010 gDNA ^1^	V.Ung v1.0 ^2^
**Number of sequences**	57,690	39,123	686
**Combined length ^3^**	568,059,011	609,523,031	519,435,864
**Minimum length ^3^**	1,000	1,000	2,922
**Average length ^3^**	9,847	15,580	757,195
**N50 length ^3^**	17,952	36,693	41,684,185
**Maximum length ^3^**	150,032	347,074	65,292,630

1. Genomic DNA assembled contigs (gDNA) 2. *Vigna unguiculata* v1.0, NSF, UCR, USAID, DOE-JGI, http://phytozome.jgi.doe.gov/ 3. Sequence lengths are in basepairs (bp)

**Table 2.** Proportion of filtered DNA paired-end reads that uniquely align to the assembled genomic DNA sequence sets from IT97K-499-35 and IT86D-1010, and to the *Vigna unguiculata* genome v1.0 assembly of IT97K-499-35. IT86D-1010 and IT97K-499-35 are the genome contig assemblies generated in this resource. Alignments were accepted if they were unique pair-end alignments within 1 kilobase of each other, with auto end-trimming of reads where required, and up to 3 mismatches per 100 base pairs.

Raw read set	IT86D-1010	IT97K-499-35	V.Ung v1.0 ^1^
**IT86D-1010 gDNA ^2^**	72.6%	64.8%	64.8%
**IT97K-499-35 gDNA**	62.5%	68.1%	65.9%

1. *Vigna unguiculata* v1.0, NSF, UCR, USAID, DOE-JGI, http://phytozome.jgi.doe.gov/ 2. Genomic DNA assembled contigs (gDNA)

**Table 3.** Proportion of assembled DNA and tissue-specific transcript contigs that align to the *Vigna unguiculata* v1.0 reference genome at three thresholds of overlap.

	Query sequences	50% ^1^	70% ^1^	90% ^1^
**IT86D-1010 gDNA ^2^**	131,241	98.0%	91.1%	65.4%
**IT97K-499-35 gDNA**	57,690	98.2%	87.8%	60.1%
**IT86D-1010 Leaf-tr**	73,278	87.7%	80.3%	56.8%
**IT86D-1010 PMG ^3^-tr ^4^**	36,179	90.7%	83.9%	59.8%
**IT86D-1010 MMC ^5^-tr**	36,058	91.8%	84.5%	60.1%
**IT86D-1010 FMT ^6^-tr**	40,158	92.2%	87.0%	66.2%
**IT86D-1010 MFG ^7^-tr**	37,710	91.4%	86.6%	65.5%
**IT86D-1010 ES ^8^-tr**	38,623	91.5%	86.6%	65.8%
**IT97K-499-35 Leaf-tr**	73,967	88.8%	81.9%	59.9%
**IT97K-499-35 PMG-tr**	35,503	91.8%	85.3%	61.9%
**IT97K-499-35 MMC-tr**	41,783	92.5%	86.0%	64.7%
**IT97K-499-35 FMT-tr**	41,580	92.0%	85.7%	64.1%
**IT97K-499-35 MFG-tr**	36,592	92.4%	87.8%	68.0%
**IT97K-499-35 ES-tr**	37,470	92.9%	88.0%	67.8%

1. Minimum overlap of query contig required within the target reference genome *Vigna unguiculata* v1.0. 2. Genomic DNA contigs (gDNA) 3. Pooled male gametophyte (PMG) 4. Transcript contigs (tr) 5. Megaspore mother cell stage (MMC) 6. Female meiotic tetrads (FMT) 7. Mature female gametophyte (MFG) 8. Early seeds (ES)

**Table 4.** Details of predicted coding gene sequences with 300bp minimum length predicted by Augustus within the assembled genomic DNA contig sets from IT97K-499-35 and IT86D-1010. Matches to NCBI’s ‘nr’ protein sequence database found through ‘blastp’ of translated predicted genes, with an e-value threshold of 1e-50.

Augustus ^1^ predicted genes	IT97K-499-35 gDNA ^2^	IT86D-1010 gDNA ^2^
**Number of predicted CDS ^3^**	61,195	62,963
**Combined length ^4^**	81,479,968	87,223,042
**Minimum length ^4^**	300	300
**Average length ^4^**	1,331	1,385
**N50 length ^4^**	1,791	1,887
**Maximum length ^4^**	14,583	15,909
**Number with ‘nr’ ^5^ match**	41,874	43,253
**Percentage with ‘nr’ ^5^ match**	68%	69%

1. Augustus *in-silico* gene prediction ( bioinf.uni-greifswald.de/augustus/; Stanke & Waack, 2003) 2. Genomic DNA assembled contigs (gDNA) 3. Coding DNA Sequence (CDS) 4. Sequence lengths are in basepairs (bp) 5. NCBI ‘nr’ database downloaded 8 ^th^ August 2017

### Leaf and reproductive cell-type and seed transcriptomes and genomic comparisons

RNA sequencing of the six tissue transcriptomes for each variety generated read counts varying from 125 to 265 million pair-end sequences. These could be assembled into transcript sets varying in size between 35,000 to 74,000 transcript contigs averaging 1 kilobase in length ( Table 5 and Table 6). In both cowpea varieties, leaf transcriptomes were the largest in terms of *de novo* assembled contig numbers and the anther transcriptomes were the smallest. In subsequent analyses RNA sequence read alignment to predicted gene models within the assembled genome resources were used to compare expression counts across tissues. The assembled genome resources for both cowpea varieties provided good coverage for the analysis of RNA sequence reads as approximately 70% of reads across all tissues could be aligned uniquely to all three genomic resources. Transcriptomes derived from IT86D-1010 displayed slightly greater alignment to the IT86D-1010 genomic resource, than the corresponding comparisons for IT97K-499-35 ( Table 7). The majority of transcript contigs (80 to 88%) across all tissues in both cultivars could be mapped to the *V. unguiculata* v1.0 reference genome with a minimum of 70% contig coverage ( Table 3). The remaining unmapped percentage could represent a range of scenarios including IT86D-1010 specific contigs, missing regions in the *V. unguiculata* v1.0 reference genome, tissue-specific extensions to the IT97K-499-35 resource or misassembled transcript contigs. Predicted gene models were considered expressed if they accrued at least 20 uniquely aligning RNASeq reads. In all tissues, approximately 30% of predicted gene models ( Table 8) showed expression and 6% of predicted gene models displayed strong tissue-specific expression. We found that on average 90% of IT86D-1010 transcript contigs could be mapped within a IT86D-1010 genomic contig and that the median genomic contig size was 67 kb relative to median transcript contig size of 1.3 kb. This indicates that this resource contains substantial amounts of genomic sequence context around expressed genes in these tissues. This will be important for future explorations of *cis*-regulatory regions associated tissue-specific gene expression.

**Table 5.** Details of assembled tissue-specific polyA RNA sequence sets from IT86D-1010. Assembled contigs of less than 300 base pairs were excluded in this analysis.

IT86D-1010	Leaf-tr ^1^	PMG ^2^-tr	MMC ^3^-tr	FMT ^4^-tr	MFG ^5^-tr	ES ^6^-tr
**Number of sequences**	73,278	36,179	36,058	40,158	37,710	38,623
**Combined length ^7^**	68,247,480	40,853,458	42,326,934	43,555,218	41,562,341	41,760,972
**Minimum length ^7^**	300	300	300	300	300	300
**Average length ^7^**	931	1,129	1,174	1,085	1,102	1,081
**N50 length ^7^**	1,208	1,602	1,660	1,494	1,538	1,501
**Maximum length ^7^**	14,930	12,310	12,441	12,276	11,392	12,272

1. Transcript contigs (tr) 2. Pooled male gametophyte (PMG) 3. Megaspore mother cell stage (MMC) 4. Female meiotic tetrads (FMT) 5. Mature female gametophyte (MFG) 6. Early seeds (ES) 7. Sequence lengths are in base pairs

**Table 6.** Details of assembled tissue-specific polyA RNA sequence sets from IT97K-499-35. Assembled contigs of less than 300 base pairs were excluded in this analysis.

IT97K-499-35	Leaf-tr ^1^	PMG ^2^-tr	MMC ^3^-tr	FMT ^4^-tr	MFG ^5^-tr	Seed ^6^-tr
**Number of sequences**	73,967	35,503	41,783	41,580	36,592	37,470
**Combined length ^7^**	69,053,233	40,224,171	46,244,665	45,725,500	39,970,331	41,525,557
**Minimum length ^7^**	300	300	300	300	300	300
**Average length ^7^**	934	1,133	1,107	1,100	1,092	1,108
**N50 length ^7^**	1,223	1,619	1,565	1,557	1,528	1,547
**Maximum length ^7^**	13,965	12,238	12,960	13,799	12,605	16,435

1. Transcript contigs (tr) 2. Pooled male gametophyte (PMG) 3. Megaspore mother cell stage (MMC) 4. Female meiotic tetrads (FMT) 5. Mature female gametophyte (MFG) 6. Early seeds (ES) 7. Sequence lengths are in base pairs

**Table 7.** Proportion of filtered raw RNASeq paired-end reads that uniquely align to the assembled genomic DNA sequence sets from IT97K-499-35 and IT86D-1010, and to the *Vigna unguiculata* genome v1.0 assembly of IT97K-499-35. Alignments by ‘biokanga align’, with up to 3 substitutions per 100 base pairs, paired-ends retained within 1 kilobase of each other and auto end-trimming of reads where required.

Raw read set	IT86D-1010	IT97K-499-35	V.Ung v1.0 ^1^
**IT86D-1010 Leaf-tr ^2^**	69.2%	68.6%	68.2%
**IT86D-1010 PMG ^3^-tr**	72.6%	72.1%	70.7%
**IT86D-1010 MMC ^4^-tr**	73.5%	72.7%	72.3%
**IT86D-1010 FMT ^5^-tr**	71.4%	70.7%	70.1%
**IT86D-1010 MFG ^6^-tr**	73.3%	72.7%	72.3%
**IT86D-1010 ES ^7^-tr**	71.3%	70.7%	70.3%
**IT97K499-35 Leaf-tr**	66.6%	67.2%	66.7%
**IT97K-499-35 PMG-tr**	69.2%	70.0%	68.5%
**IT97K-499-35 MMC-tr**	69.9%	70.4%	69.9%
**IT97K-499-35 FMT-tr**	69.3%	69.8%	69.3%
**IT97K-499-35 MFG-tr**	69.7%	70.1%	69.6%
**IT97K-499-35 ES-tr**	68.4%	68.8%	68.1%

1. *Vigna unguiculata* v1.0, NSF, UCR, USAID, DOE-JGI, http://phytozome.jgi.doe.gov/ 2. Transcript contigs (tr) 3. Pooled male gametophyte (PMG) 4. Megaspore mother cell stage (MMC) 5. Female meiotic tetrads (FMT) 6. Mature female gametophyte (MFG) 7. Early seeds (ES)

**Table 8.** Proportion of predicted gene models that accrue RNA sequencing reads. Counts shown for gene models with more than 20 uniquely aligning RNASeq reads.

Transcriptome	August Gene Models ^1^ Expressed	Proportion of total gene models
**IT86D-1010 Leaf-tr ^2^**	21,024	31%
**IT86D-1010 PMG ^3^-tr**	21,315	31%
**IT86D-1010 MMC ^4^-tr**	20,672	31%
**IT86D-1010 FMT ^5^-tr**	21,356	32%
**IT86D-1010 MFG ^6^-tr**	20,290	30%
**IT86D-1010 ES ^7^-tr**	20,486	30%
**IT97K-499-35 Leaf-tr**	21,088	31%
**IT97K-499-35 PMG-tr**	20,953	31%
**IT97K-499-35 MMC-tr**	20,905	31%
**IT97K-499-35 FMT-tr**	20,274	30%
**IT97K-499-35 MFG-tr**	20,005	30%
**IT97K-499-35 ES-tr**	20,871	31%

1. Augustus *in-silico* gene prediction on IT86D genomic contigs ( bioinf.uni-greifswald.de/augustus/; Stanke & Waacke, 2003) 2. Transcript contigs (tr) 3. Pooled male gametophyte (PMG) 4. Megaspore mother cell stage (MMC) 5. Female meiotic tetrads (FMT) 6. Mature female gametophyte (MFG) 7. Early seeds (ES)

## Data availability

All data associated with this publication are provided on the Commonwealth Scientific and Industrial Research Organisation (CSIRO) Data Access Portal ( http://data.csiro.au). Data are available at the direct link: https://doi.org/10.4225/08/5b1723666d6a5 ( Spriggs *et al.*, 2017).

Data are released publicly under the Creative Commons Attribution 4.0 International License ( CC BY 4.0).

## Supplementary material

Supplementary Figure 1: Pedigree maps of IT97K-499-35 and IT86D-1010.
