# Supplementary material for: Assembled genomic and tissue-specific transcriptomic data resources for two genetically distinct lines of Cowpea ( Vigna unguiculata (L.) Walp)
Source: Gates Open Res. 2018 Jun 18;2:7. Originally published 2018 Feb 9. [Version 2] doi: 10.12688/gatesopenres.12777.2 (PMC5841572; doi:10.12688/gatesopenres.12777.2)
Supplement: Supplementary file 1 [file gatesopenres-2-13907-s0000.tgz › 83e2a033-9125-4058-b5a3-5d44b3c7c74f.pdf]

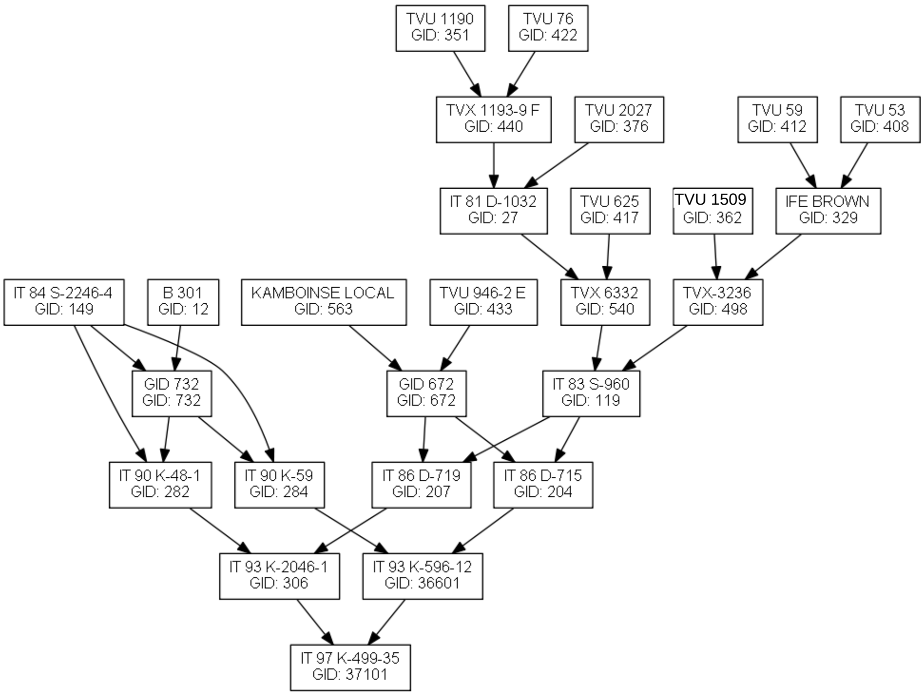

A. Pedigree map of IT97K-499-35

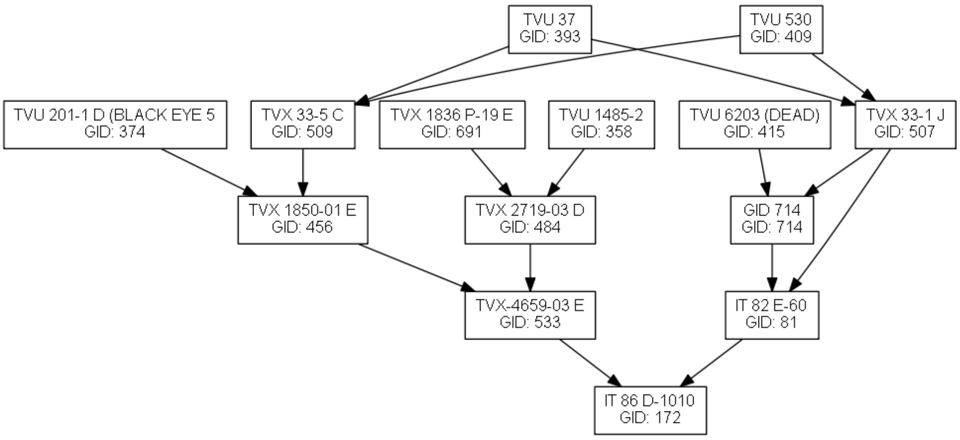

B. Pedigree map of IT86D-1010

Supplementary Figure 1: Pedigree maps of IT97K-499-35 and IT86D-1010. Source: BB Singh and Ousmane Boukar, International Institute of Tropical Agriculture: Pers comm.
