# Supplementary material for: Assembled genomic and tissue-specific transcriptomic data resources for two genetically distinct lines of Cowpea ( Vigna unguiculata (L.) Walp)
Source: Gates Open Res. 2018 Jun 18;2:7. Originally published 2018 Feb 9. [Version 2] doi: 10.12688/gatesopenres.12777.2 (PMC5841572; doi:10.12688/gatesopenres.12777.2)
Supplement: Peer review history

# Peer review: Referee response for version 1

**DOI:** 10.21956/gatesopenres.13837.r26246

**Published:** 2018-03-07

**Authors:** Timothy J. Close

**Affiliations:** 1 Department of Botany and Plant Sciences, University of California, Riverside, Riverside, CA, USA

The authors have produced genomic and transcriptomic sequences and assemblies from two cowpea accessions that have been possible to transform, IT97K-499-35 and IT86D-1010. They have used this information to develop annotated gene sequences. This is a useful contribution of information on the cowpea genome, adding significantly to the body of knowledge that has been developed from other cowpea genome sequencing and transcriptome efforts, of which there are only a few. They made good use of the IT97K-499-35 reference genome that became available in 2017. The biological and bioinformatic methods are sufficiently explained and seem appropriate. The various tables indicate a consistent level of quality across samples. I have just a few questions or comments.

1. I am somewhat confused by the consistency of the proportion of total gene models that accrue RNA sequencing reads (Table 8) versus the statement on page 5 that leaf transcriptomes had the largest number of contigs and anthers the smallest. I must not be grasping the counting methods. Please clarify.
2. Plant materials and tissue collection. Were these two lines/accessions propagated by single seed descent, such that one would expect every plant within each line/accession to be genetically identical or nearly so? Or was more than on plant taken forward at each generation? Single seed descent for many generations accomplishes homozygosity, providing a single haplotype, which simplifies sequence assembly and alignments. If records are available to address this, then please add that information.
3. Table 3. The values in the 90% column all are considerably lower than the 50% and 70% columns. Is there a simple explanation for this?
4. We received IT97K-499-35 from Mike Timko, who had previously received it from IITA. We took it through three rounds of SSD before bulking and, fortunately, found that the plant used to establish the bulk seed was 100% homozygous. Please adjust the wording on p.4 to indicate that Mike Timko was our source of this accession.
5. JGI annotated the pseudomolecules that were developed at UC Riverside.

I have read this submission. I believe that I have an appropriate level of expertise to confirm that it is of an acceptable scientific standard.

# Peer review: Referee response for version 1

**DOI:** 10.21956/gatesopenres.13837.r26245

**Published:** 2018-03-01

**Authors:** Ian D. Godwin

**Affiliations:** 1 School of Agriculture and Food Sciences, University of Queensland, Brisbane, Qld, Australia

This is a useful note on the production and assembly of genomic and transcriptomic data for two lines of cowpea. Like most "resource" papers, the manuscript is not particularly exciting or engaging reading, but that is not really the point. The point is to provide the data and allow interested users to access the data and share results. In my opinion, it can help readability to at least include something of biological interest in such a paper, such as a small case study interpreting the data, however, this is not necessary for the paper to stand on its own scientific merits.

 Some minor comments.

1. The way I read the abstract, it appears that IT86D-1010 is the second variety and by way of disctinction, that it is transformable. However, in the Introduction, it is made clear that both lines are transformable. The Abstract should be modified to improve the clarity of this.
2. Page 5 para 2: varyingin ... varying in
3. Final paragraph under Data Availability: Data is .... Data are

I have read this submission. I believe that I have an appropriate level of expertise to confirm that it is of an acceptable scientific standard.

# Peer review: Referee response for version 1

**DOI:** 10.21956/gatesopenres.13837.r26247

**Published:** 2018-02-28

**Authors:** Valerie Hecht

**Affiliations:** 1 School of Plant Science, University of Tasmania, Hobart, Tas, Australia

The Data Note “Assembled genomic and tissue-specific transcriptomic data ressources for two genetically disctinct lines of Cowpea ( *Vigna unguiculata* (L.) Walp)” by A. Spriggs and co-workers describes the obtention of genomic and transcriptomic data from two Cowpea varieties, IT97K-499-35 and IT86D-1010.

 While an unclustered genome assembly is already publicly available for Cowpea ( *Vigna unguiculata* v1.1; https://phytozome.jgi.doe.gov/pz/portal.html), the genomic dataset presented in this Data Note for two distinct varieties of Cowpea increases the sequence availability for this species.

 The transcriptomics data obtained mainly focusses on reproductive tissues, including anthers and ovules from dissected floral buds at different stages. The five reproductive stages used for RNAseq are pooled male gametophyte (PMG), megaspore mother cell (MMC), female meiotic tetrad (FMT), mature female gametophyte (MFG) and early developing seeds (ES) containing young developing embryos. In addition to these, young unexpanded leaves were also used. All those samples were sequenced in triplicates for both varieties of cowpea, except MMC and FMT from IT97K-499-35 where only two replicates were sequenced. These six tissue specific datasets per variety will be a very useful ressource for differencial expression analysis in reproductive studies of Cowpea.

 The rationale to create the datasets is clearly and well explained in the introduction section. The methods and protocols used could be expanded as detailed below. The datasets are accessible though CSIRO Data Access portal, and are presented appropriately in the article as data analysis summaries in tables.

 Comments:

- Table 5 and 6 show “details of the assembled tissue-specific polyA RNA sequences” from both varieties, but the nucleic acid extraction section of the Methods does not mention polyA RNA isolation. If polyA RNA was obtained from each tissue, the protocol used for it should be detailed in the methods.
- There are no details about the genomic and cDNA library constructions. Even if those were done commercially by AGRF, details on how the libraries were made should be provided.
- The analysis of the raw sequences was done using Biokanga, a CSIRO developed suite of Next Generation Sequencing analysis tools. Some information about the quality control analysis of the reads obtained for each library should be presented (Basic statistics, Per base and per sequence quality scores, GC content, Miscalled bases…) in order to evaluate the quality of the raw data.

I have read this submission. I believe that I have an appropriate level of expertise to confirm that it is of an acceptable scientific standard.
